# Some Observations on the Use of Copper Amalgam

**Published:** 1892-07

**Authors:** J. Allen Osmun

**Affiliations:** Newark, N. J.


### SOME OBSERVATIONS ON THE USE OF COPPER AMALGAM.[1]

BY DR. J. ALLEN OSMUN, NEWARK, N. J.

Mr. President and Gentlemen,—With the invitation to present a paper here to-night also came the suggestion that its subject should be copper amalgam.

If I had followed my own option in the selection of a subject I think I should have chosen another theme for our consideration, not because that there is not an abundant field open for thought and investigation. No one, I take it, has the assurance to pretend that copper amalgam is the universal plastic filling-material, although, only a very short time ago, it certainly looked as if this claim would be made; and, on the other hand, to make the assertion that it is without merit is, to my mind, equally absurd.

The literature of copper amalgam is quite meagre, although every now and again some one comes forward who says he used it many years ago. But to all practical ends it is a comparatively new agent for filling teeth, and it was not until the summer of 1887, I think that is the year, on the appearance of an article published in the *Independent Practitioner*, by Dr. George H. Weygant, of Canada, that the attention of the dental profession was arrested and the general use of this material for fillings adopted.[2] My own use dates from this time. At first I was very sceptical as to its merits, but, upon using and watching it carefully, became convinced that, for a certain class of teeth and for buccal and approximal cavities, it possessed undoubted merits. Dr. Weygant says that he searched diligently through all the dental literature available to obtain information upon this subject. Reports of societies, correspondences, chemistries, encyclopædias, scientific works, were silent upon it. I can substantiate all this, because I have searched in vain for light, and could not find anything of much interest bearing on it. Although copper as an ingredient of amalgams has long been recognized, and testimony varies as to its good and bad qualities in such combinations, it has always been a source of wonder to me that it was received with so much favor, and can only account for it by the fact that in the class of teeth I

---

[1] Read at a meeting of the New York Odontological Society, April 19, 1892.

[2] Copper amalgam was employed as a filling-material in Germany in 1879. Its use or manufacture did not originate in this country.—[Ed].

have in mind and the kind of cavities just mentioned, other amalgams have been found thoroughly unreliable, and when the report got abroad that copper amalgam neither shrank nor bulged, and that after months of use showed perfect margins, it was received with joy.

Copper amalgam has received since its introduction the most extravagant praise and the most severe denunciation. I am not prepared to admit that the goodness comes from an innate righteousness, or the badness from total depravity; or, on the other hand, that all its peculiar conduct is caused by its environment, but am fully persuaded that there is a principle involved, which, if it could only be discovered, would overcome all of its vagaries.

Dr. W. B. Ames, of Chicago, in a paper read before the Mississippi Valley Dental Society, and published in the *Dental Review* of April, 1891, takes the position that he is of the opinion that to too much grinding, while in the process of manufacture and by the operator before placing the mass in the cavity, is due largely the cause of the washing or cupping out of amalgam fillings. He attributes this fact to be largely caused by the establishment of an electric current acting on the free copper. While giving Dr. Ames great credit for his conclusions as to the theory of the electric chemical action, I am not fully clear in my own mind that this can be the cause, but only a factor, and that the cause must be looked for in the manipulation, and that grinding the mass is not the reason for failure in the use of copper amalgam.

To the more full comprehension of my theory as regards the peculiar conduct of this filling-material I desire to recapitulate and group together the more prominent and peculiar results observed in the different mouths that have come under my own eyes.

1. We all have seen cases that have filled us with admiration, black but clean fillings, hard as adamant, and edges absolutely perfect,—the ideal plastic filling.

2. Then, again, we have observed fillings, black, but dirty in appearance, teeth in the immediate neighborhood all discolored with the stain of the disintegrated filling, probably the black sulphuret of mercury, referred to in Dr. Ames's paper,—however, saving the teeth as far as recurrence of decay.

3. The third class of fillings is very much like the preceding, only with this difference: that at the cervical edge we find that the filling has decomposed, and in some cases entirely wasted, so that it does not afford the tooth any protection; in fact, it resembles, to a great extent, the phenomenon observed with the oxyphosphate fillings, and leads one to the conviction that the same agent is responsible for this condition of affairs. This I consider one of the most deplorable situations that can possibly exist. Much more so than the other which is so familiar, and has brought copper amalgam fillings into such disrepute, and which I assign to the fourth class.

4. Where we find the filling-material of a light- or dull-gray color softened, and in some cases to such an extent as to be easily removed with an excavator, while in others it simply is washed away, leaving a hard, glassy appearance, yet not hard enough to resist whatever the agent may be, and slowly the fillings cup. Yet this is not so dangerous to the welfare and preservation of the tooth as that class of fillings just mentioned which give way at the cervical edge, because, in the one case, no knowledge is had until great mischief is accomplished, while in the latter case it gives way from the outside surface, and the patient is conscious that something is radically wrong and hence seeks relief.

It does not seem plausible that all of these different conditions can exist sometimes in the same mouth, which I have repeatedly seen, and the cause be attributed to the environment. Referring again to Dr. Ames's article, where he says "that it is unreasonable to suppose that there was ever a condition of the saliva sufficiently acid to dissolve copper and mercury to the extent we often see, . . . unless the action was in some way intensified,—as these metals are only soluble in nitric acid, and the more powerful nitro-muriatic," and then goes on to show that the current is established by the environment and the metals setting up a continuous current that dissolves the filling.

This theory presents a very plausible and enticing solution of the question, and yet I cannot see my way to fully endorse or accept it, because in so many cases in the same mouth and, so far as can be ascertained, with exactly the same conditions as to location of filling, with the same character of saliva, we find the "very, very good" and the "horrid." I am of the opinion that the cause must be looked for in another direction.

It has been generally accepted that the agent that decomposed these fillings is an acid; yet in many tests made with litmus-paper I have repeatedly found an alkaline reaction, and in many mouths, that were intensely acid, we have observed perfect fillings. Dr. Ames, in a personal letter received a short time since, admits that he has also observed this peculiar condition. This is one of the difficult problems to solve, for if we always found that copper amalgam fillings decomposed in the mouth in acid fluids, or always in alkaline surroundings, then we could perhaps sooner find a solution. This leads me to come to the conclusion that the cause must be looked for in some other direction,—possibly that the environment may be a predisposing, but not the real cause.

I have not been able to get any information upon the results obtained in the use of "Sullivan's cement." I have written to many persons, but have not up to the present had any light thrown upon it. It has been universally admitted that it was fearfully dirty to use, and that it not only discolored itself,—that is, the filling,—but that it turned the tooth almost as black as the filling; but I cannot find out to a certainty about the decomposition of the filling-material. Perhaps we shall have this point of difficulty settled here to-night in the discussion.

I have long contended that the fault of all these different conditions could be laid to either imperfect methods of manufacturing, or some defect in preparing the material by the operator.

As to the methods of manufacturing, I am not well enough informed, or have not had an experience which would justify an expression of opinion; but I have a positive judgment as to the preparation of the material by the operator. My experience has been confined almost exclusively to Weygant's, Ames's, and the S. S. White, and, to some extent, to Russell's productions. I find but little difference in the working qualities of these different varieties, and I think there is but little in the length of time that they last. In the first use of copper amalgam by the profession, the one great point was to get the material to solidify in the cavity within a reasonable time. Heat and rubbing were recognized as the important agents used to obtain this end, and it was soon recognized that the more heat applied the longer it took to become hard. Then it became apparent that there was too much mercury used, and an effort was made by many manufacturers to produce a dryer amalgam. I presume, in compliance with a feeling expressed by a friend of mine, who said he would rather buy his mercury at a less price than was asked for copper amalgam, Dr. Ames put upon the market, as well as Dr. Russell, a dry copper amalgam. It had the great fault that in applying heat sufficient to make it work properly it was extremely likely to be burned and oxidized, and rendered thoroughly unfit for use. We want sufficient mercury in it, so that when it is heated it will soften without fear of destroying its chemical relation. Some very peculiar experiences were had in the use of this material when it came into general use. One gentleman told me that he put in a filling early in the morning, and that he examined it late in the afternoon, and he found that it was just as he had left it, and so far as he could ascertain it had not hardened at all. Now, we all admit that this was produced by too much mercury being left in the material, with the application of too much heat.

I am compelled again to quote Dr. Ames, simply from the fact that I regard him as a most careful and thorough investigator, and it does seem strange that his conclusions should be so entirely different from those I am endeavoring to present to you to-night from my stand-point. He says that he has become convinced "that all or any grinding was bad for the material, . . . and that as little heat be used as is possible to get it into shape to be placed in the cavity." Dr. Weygant also makes this same observation, while another gentleman states that the material having commenced to "set" before being placed in the cavity, is the reason for the cause of failure of the filling. My own experience has been quite the opposite of this. I have had far better results when I heated the material a good deal, given it thorough trituration, and removing all the mercury possible, leaving the material so it could be worked. In fact, those fillings that have stood the very best, where the material has left a stain on my hand while working it, resembling copper in color, which once seen can always be recognized.

I have therefore come to the conclusion that for the successful working of copper amalgam we must have the material as free as possible of mercury, as this is unquestionably only a solvent, having no strength in itself, and any excess weakening the material. It may be true, as Dr. Ames has so ably pointed out, there is a current established in the mouth by the presence of weak acids, and this may be the agent that produces the mischief; but, nevertheless, we can readily see that this is not always the case, for, as has been already mentioned, in the same mouth, with the same conditions as to weak acids, the contiguous position of other kinds of fillings, crowns, etc., do not present the same characteristics; but the fact is, that in many such mouths you find good, bad, and indifferent fillings, so I think that, as a gentleman known somewhat to fame once said, "this is not a theory but a condition that confronts us." We can accept the sentiment in the case of copper amalgam.

I have corresponded with a number of well-known practitioners, and their experiences seem to justify the conclusion that all the difficulties can be traced to improper manipulation in the preparation of the material. I fully coincide with both Dr. Ames and

Weygant, that excessive heating produces deplorable results; but that heat is the one great agent to obtain a good filling-material I am fully persuaded. Take, for instance, by way of illustration, steel; you heat it to a white heat, and you have ruined it; it is absolutely worthless. Take the same piece, heat it moderately, manipulate it by pounding it, and the more times you heat it and work it, the more tough and tenacious it becomes. My own experience in the use of copper amalgam justifies this theory. I have taken out fillings of this material that were soft and of no value in saving the teeth, and put right back in the same cavities with the same surrounding conditions. If a copper amalgam filling be worked as I am endeavoring to demonstrate, heating many times, with thorough rubbing, clearing them of all excess of mercury, they will stand the test in every way, and become a thing of beauty and a joy forever. In other words, we must temper the copper that constitutes so important a factor. That copper is susceptible of tempering, I think can be demonstrated.

Another important factor in the successful working of copper amalgam, in addition to heat and rubbing and getting clear of all surplus mercury, is force in placing the material in the cavity. There must be much burnishing and force used in working this amalgam, and to the want of it, I think, can be attributed to a certain degree some of the failures which occur at the cervical edge just referred to; and in addition to all this, to have the cavity as free from moisture as possible. Copper amalgam has great affinity for moisture, and while it has been claimed that it will save a tooth even if put in under water, the fact remains that the safety and permanence of that filling would be better assured if worked dry. Then, again, for the successful working of copper amalgam one must use discretion and judgment. It is not a panacea for all the cavities that come under our observation, neither does it offer a premium on defective preparation of the cavity, or slothful methods of insertion. Gold is admitted by all as the royal filling-material, yet no operator of to-day claims that it is good practice to use it in all classes of teeth, or in all the cavities that present themselves for treatment. Yet we often hear this question asked, "What amalgam do you use?" implying by the question, do you find one amalgam that will fit all kinds and conditions. Copper amalgam, from my experience, justifies me in coming to the conclusion that there is a subtle principle involved somewhere, either in the manufacture or in the manipulation by the operator, that produces all of the various manifestations, and for this reason, if we found all of the fillings in a certain mouth in exactly the same condition, and the fillings in another mouth bearing the same relation to each other, then we could, I think, very appropriately assign the cause to environment. I do not want to be understood as claiming that environment plays no part in the disintegration of the fillings, but that it is only one factor, helped on by an improper preparation of the material. I am fully persuaded that this filling has many good qualities to recommend it to our favor; but that it must be used carefully and with good judgment admits of no argument.

---

